# Altered glutamine metabolism in platinum resistant ovarian cancer

**DOI:** 10.18632/oncotarget.9317

**Published:** 2016-05-12

**Authors:** Chantelle D. Hudson, Alyssa Savadelis, Anil Belur Nagaraj, Peronne Joseph, Stefanie Avril, Analisa DiFeo, Norbert Avril

**Affiliations:** ^1^ Department of Radiology, Case Center for Imaging Research, Case Western Reserve University, Cleveland, OH 44106, USA; ^2^ Case Comprehensive Cancer Center, Case Western Reserve University, Cleveland, OH 44106, USA; ^3^ Department of Pathology, Case Western Reserve University, Cleveland, OH 44106, USA

**Keywords:** ovarian cancer, cisplatin resistance, glutamine metabolism, glutaminase inhibitors

## Abstract

Ovarian cancer is characterized by an increase in cellular energy metabolism, which is predominantly satisfied by glucose and glutamine. Targeting metabolic pathways is an attractive approach to enhance the therapeutic effectiveness and to potentially overcome drug resistance in ovarian cancer. In platinum-sensitive ovarian cancer cell lines the metabolism of both, glucose and glutamine was initially up-regulated in response to platinum treatment. In contrast, platinum-resistant cells revealed a significant dependency on the presence of glutamine, with an upregulated expression of glutamine transporter ASCT2 and glutaminase. This resulted in a higher oxygen consumption rate compared to platinum-sensitive cell lines reflecting the increased dependency of glutamine utilization through the tricarboxylic acid cycle. The important role of glutamine metabolism was confirmed by stable overexpression of glutaminase, which conferred platinum resistance. Conversely, shRNA knockdown of glutaminase in platinum resistant cells resulted in re-sensitization to platinum treatment. Importantly, combining the glutaminase inhibitor BPTES with platinum synergistically inhibited platinum sensitive and resistant ovarian cancers *in vitro*. Apoptotic induction was significantly increased using platinum together with BPTES compared to either treatment alone. Our findings suggest that targeting glutamine metabolism together with platinum based chemotherapy offers a potential treatment strategy particularly in drug resistant ovarian cancer.

## INTRODUCTION

Epithelial ovarian cancer is the most common type of ovarian malignancy and often diagnosed in advanced stages of disease. The standard treatment includes cyto-reductive surgery followed by systemic platinum and taxane-based chemotherapy [1]. The majority of women who initially respond to chemotherapy relapse after first-line treatment and will ultimately develop resistance to platinum based chemotherapy [2]. The development of drug resistance contributes to a poor outcome of patients with advanced stage ovarian cancer [3, 4]. Therefore, it is important to understand the mechanisms involved with the development of drug resistance, in order to identify new therapeutic approaches.

Like most cancers, ovarian cancer is dependent on the increased utilization of energy sources to maintain ATP levels and intermediates required for enhanced cellular metabolism, cell growth and proliferation [5]. Glucose and glutamine are two main energy sources for cancer cells [6]. Glucose is metabolized via increased aerobic glycolysis, and the increased glycolytic flux to lactate results in decreased pyruvate availability for the mitochondrial tricarboxylic acid (TCA) cycle. Recent evidence demonstrates that a number of malignant tumors are glutamine dependent to maintain their energy requirements [7]. A critical step in the utilization of glutamine is its conversion to glutamate by the mitochondrial enzyme glutaminase (GLS). Glutamate and glutamate-derived metabolites can serve as intermediates to supply the TCA cycle, generate glutathione and contribute to fatty acid production [8, 9].

The MYC oncogene encodes the transcription factor c-Myc which controls the expression of multiple key genes involved in the regulation of metabolic pathways including glycolysis and glutaminolysis [10]. Given the central role of c-Myc in regulating metabolic pathways as well as cellular growth it is a commonly amplified oncogene in many hematologic and solid malignancies, including epithelial ovarian cancer [11, 12]. However, the precise regulation of glutamine metabolic pathways in cancers and its association with c-Myc is not fully understood and little is known on the role of glutamine metabolism in the development of drug resistance in ovarian cancer. Recent studies have highlighted the role of glutamine metabolic pathways in ovarian cancer progression and high glutaminase (GLS) expression in human tissue samples has been shown to be associated with poor survival [13–15].

Much emphasis has been directed to the development of glutaminase (GLS) inhibitors [16]. In pre-clinical studies, inhibition of glutaminase (GLS) by either siRNA or via the allosteric inhibitor, bis-2-(5-phenylacetamido-1,2,4-thiadiazol-2-yl)ethyl sulfide (BPTES) delayed the growth of various hematologic and solid tumors *in vitro* [17–21] and *in vivo* [16, 17, 22, 23]. However, single agent treatments with metabolic pathway inhibitors are unlikely to be curative, due to adaptive mechanisms involving a switch in energy sources in cancer cells.

In the present study, we further explored the role of glutamine metabolism during platinum based treatment of drug sensitive and resistant ovarian cancer. We identified c-Myc as the upstream regulator increasing the dependency of platinum resistant ovarian cancer cell lines on glutamine metabolism via the TCA cycle and in the regulation of oxidative phosphorylation. Furthermore, we discovered that glutaminase (GLS) overexpression confers platinum resistance and its inhibition via BPTES re-sensitized platinum resistant cells. Our study demonstrates that glutamine utilization is a critical step in the development of platinum resistance in ovarian cancer and that adding inhibitors of glutamine metabolic pathway may be beneficial in the treatment of ovarian cancer patients.

## RESULTS

### Increased glutamine utilization during cisplatin treatment

To investigate changes in glucose and glutamine utilization we assessed the uptake of radiolabeled [C-14]deoxyglucose ([C-14]DG) and [H-3]glutamine ([H-3]GLN) during cisplatin treatment. We evaluated two paired cell lines: the cisplatin sensitive A2780 cell line and its cisplatin resistant derivative CP70, together with the cisplatin sensitive OV81.2 cell line, which is a primary cell line derived from a high grade serous ovarian cancer patient. The cisplatin resistant derivative OV81.2-CP10 (referred to as CP10 henceforth) was derived by propagating OV81.2 cells in the presence of cisplatin for 10 passages thus selecting for resistant clones [24].

**Figure 1:**
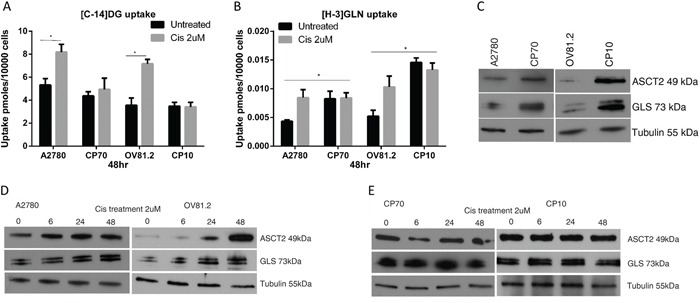
Cisplatin resistant cells are glutamine dependent **A** and **B.** [C14]-2DG and [H-3]GLN uptake in ovarian cancer cells with and without cisplatin treatment (2uM), normalized to cell number. (A) Increased [C14]-DG uptake was observed in cisplatin surviving A2780 and OV81.2 cells after 48 hr which was not observed in the cisplatin resistant CP70 and CP10 cell lines. No further increase in tracer uptake is found when the resistant cell lines are treated with cisplatin. (B) Baseline [H3]GLN uptake is 2-fold higher in the cisplatin resistant CP70 compared to A2780 and 3-fold higher in CP10 cells compared to OV81.2 cells. GLN uptake is increased in the sensitive but not the resistant cell lines after 48 hr cisplatin treatment (p<0.01). Experiments were performed in triplicate and repeated 3 times. Uptake is normalized to cell number. Graphs represent mean (boxes) and SD (bars; n=9). **C.** Western blot showing increased glutamine transporter ASCT2 and glutaminase (GLS) expression in CP70 and CP10 cells compared to the sensitive A2780 and OV81.2, respectively (p< 0.01) **D, E.** Western blot showing increasing levels of GLS and ASCT2 protein in response to cisplatin treatment in sensitive cell lines, and no change in platinum resistant cells.

The baseline uptake of [C-14]deoxyglucose showed little difference between the paired cisplatin sensitive and resistant cell lines (Figure 1A), whereas the baseline uptake of [H-3]glutamine was increased 2-fold in cisplatin resistant CP70 cells compared to sensitive A2780 cells and 3-fold in cisplatin resistant CP10 cells compared to sensitive OV81.2 cells (p<0.01, Figure 1B). Interestingly, both A2780 and OV81.2 showed a 1.5 – 2-fold increase in radiolabeled [C-14]DG and [H-3]GLN uptake 48hr after start of cisplatin treatment (p<0.01; Figure 1A, 1B). In contrast, no change in glucose or glutamine uptake was observed in the cisplatin resistant cell lines CP70 and CP10 upon exposure to cisplatin (Figure 1A, 1B).

To better understand the mechanism regulating the dependence on glutamine utilization in the cisplatin resistant cell lines, we analyzed the expression of the high affinity glutamine transporter (ASCT2) and glutaminase (GLS), which converts glutamine to glutamate. Western blot analysis showed increased expression of the glutamine transporter ASCT2 and glutaminase (GLS) in cisplatin resistant cell lines compared to the sensitive cell lines (p< 0.01; Figure 1C), confirming the increased utilization of exogenous glutamine in cisplatin resistant cells. Furthermore, western blot analysis revealed increased ASCT2 and GLS expression in A2780 and OV81.2 cells early during cisplatin treatment (p<0.01, Figure 1D), which was maintained in cisplatin treated cells at 48hr (Figure 1D). The expression of ASCT2 and GLS was unaffected by cisplatin treatment in the resistant CP70 and CP10 cells, consistent with the lack of increased [H-3]GLN uptake upon cisplatin treatment (Figure 1E). These results suggest that cisplatin resistant cells have increased glutamine requirements and upon cisplatin treatment, glucose and glutamine utilization is increased in cisplatin sensitive cells as well.

### Cisplatin resistant ovarian cancer cells utilize glutamine for oxidative phosphorylation

**Figure 2:**
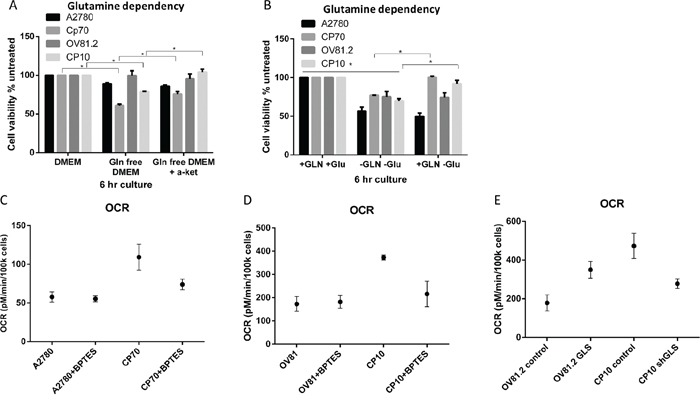
Cisplatin resistant cells depend on glutamine for oxidative phosphorylation **A.** Ovarian cancer cell lines were cultured in complete media, glutamine free or glutamine free containing α-KG (4 mM). **B.** Added GLN restores cell viability of CP70 and CP10 cells but not A2780 or OV81.2 cells. Cell viability was determined using the Vi-CELL trypan blue exclusion counter. Experiments were performed in triplicate and repeated 3 times. Viability is expressed relative to the cell line grown in complete media. Graphs represent mean (boxes) and SD (bars; n=9). **C.** A2780 or CP70 **D.** OV81.2 or CP10 cells were treated with DMSO or BPTES (50uM) for 24 hr before measuring oxygen consumption rate (OCR). OCR is increased in resistant cell lines compared to the sensitive cell lines. The decrease using BPTES (50uM) confirms the glutamine dependency of cisplatin resistant CP70 and CP10 cells. Experiments were carried out in triplicates and repeated 3 times. Graphs represent mean (dots) and SD (bars; n=9). **E.** GLS over expression in the sensitive cell line OV81.2 results in increased OCR compared to OV81.2 control (p<0.01). CP10 shGLS transduced cells have reduced OCR compared to CP10 shRNA control (p<0.01).

In order to determine the level of glutamine dependency in cisplatin resistant cells we assessed the effects of glutamine deprivation on cellular viability. We found that cisplatin resistant CP70 cells were more sensitive to glutamine deprivation showing a 40% decrease in cell viability compared to 11% in the cisplatin sensitive A2780 cell line after culture in glutamine free media over 6hr (Figure 2A). Similarly, in the human primary cell line OV81.2 cell viability was unaffected by glutamine deprivation whereas the cisplatin resistant CP10 cells showed a 21% decrease in cell viability (Figure 2A). Furthermore, the addition of glutamine after culturing in glutamine free media for 6hr reversed the effects on cell viability in the cisplatin resistant CP70 and CP10 cells but not in the cisplatin sensitive A2780 and OV81.2 cell lines (p<0.01, Figure 2B). These results suggest that the cisplatin resistant CP70 and CP10 cells have a higher glutamine dependency and rely more on glutamine metabolism for survival. α-ketoglutarate (α-KG) is the glutamine metabolite that enters the mitochondrial TCA cycle. Addition of dimethyl α-KG, a membrane permeable alpha-ketoglutarate analogue, reduced the cell death observed in the CP70 and CP10 cell lines upon glutamine deprivation (p<0.01, Figure 2A), suggesting that the cisplatin resistant cell lines depend on glutamine to feed the TCA cycle for subsequent cell survival.

The TCA cycle is directly linked to mitochondrial respiration, which we measured by assessing the oxygen consumption rate (OCR). We observed a 2-fold higher OCR in cisplatin resistant CP70 cells compared to A2780 and a 3-fold higher OCR in cisplatin resistant CP10 cells compared to OV81.2 (p<0.01, Figure 2C, 2D). The increased OCR is glutamine dependent, as blocking the glutamine metabolism using the glutaminase inhibitor BPTES (50 uM) reduced the OCR in the CP70 and CP10 cells to the level found in the corresponding platinum sensitive cell lines (Figure 2C, 2D). Furthermore, the level of OCR in the A2780 and OV81.2 cells was not reduced when blocking glutaminase via BPTES (Figure 2C, 2D) suggesting that the main energy source for mitochondrial respiration in cisplatin sensitive cell lines is via glucose derived pyruvate or fatty acids entering the TCA cycle. We confirmed a direct role of GLS and glutamine metabolism in driving mitochondrial respiration in cisplatin resistant cells by over-expressing glutaminase in OV81.2 cells (Figure 4A) or down regulating GLS expression in CP10 cells (Figure 5A) using lentivirus transfection. We found a 2-fold increase in OCR in the OV81.2 cells over-expressing glutaminase compared to control, whereas shGLS knockdown in the CP10 cells resulted in reduction of OCR compared to the control shRNA cells (p=0.01, Figure 2E).

### C-Myc regulates glutamine metabolism in cisplatin resistant cells

**Figure 3:**
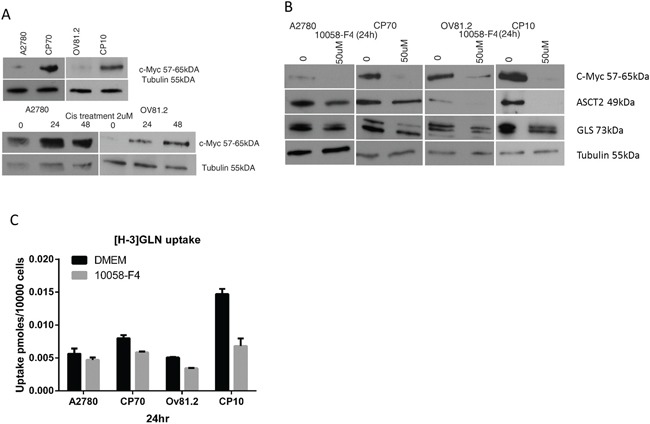
c-Myc regulates glutamine metabolism in cisplatin resistant cells **A.** Western blot showing increased c-Myc expression in the resistant cell lines compared to the sensitive cell lines as well as increasing c-Myc expression in A2780 and OV81.2 cells after 24 and 48hr of cisplatin (2uM) treatment. **B.** Western blot showing reduction of c-Myc, ASCT2 and GLS expression in A2780/CP70 and OV81.2/CP10 in response to treatment with c-Myc inhibitor 10058-F4 (50uM). **C.** C-Myc inhibition using 50uM 10058-F4 decreased [H-3]GLN uptake in all cell lines, which is more pronounced in the CP70 and CP10 cells. Uptake is normalized to cell number. Experiments were performed in triplicate and repeated 3 times. Graphs present mean (boxes) and SD (bars; n=9).

C-Myc has been shown to regulate metabolic pathways and increase the expression of glutamine transporters (ASCT2) and glutaminase (GLS) [8, 25, 26]. Western blot analysis showed increased c-Myc protein expression in cisplatin resistant CP10 and CP70 cell lines compared to the sensitive OV81.2 and A2780 cell lines, respectively (Figure 3A). In addition, we found an increasing c-Myc expression in both sensitive cell lines after 24 and 48hr cisplatin treatment (2uM) (p< 0.01, Figure 3A). To assess the direct effect of blocking c-Myc on glutamine metabolism we used the c-Myc inhibitor 10058-F4, which inhibits the c-Myc-Max interaction and prevents transactivation of c-Myc target gene expression. As c-Myc is downregulating its own expression in a feedback loop, we assessed the efficiency of 10058-F4 by reduction of c-Myc expression, which was abolished after 24hr treatment with 50uM 10058-F4 in all cell lines (P<0.01, Figure 3B). The expression of ASCT2 and glutaminase were also reduced with 50uM 10058-F4 treatment after 24hr (P<0.01, Figure 3B), indicating a functional role of c-Myc in regulating glutamine uptake and utilization. Furthermore, inhibiting c-Myc in both, CP10 and CP70 cell lines had a greater effect on reducing glutamine uptake compared to the cisplatin sensitive OV81.2 and A2780 cell lines (P<0.01, Figure 3C). Our results suggest that the increased glutamine uptake in platinum resistant cell lines is at least in part mediated by the increased expression of c-Myc in these cell lines.

### Expression of glutaminase contributes to cisplatin resistance

**Figure 4:**
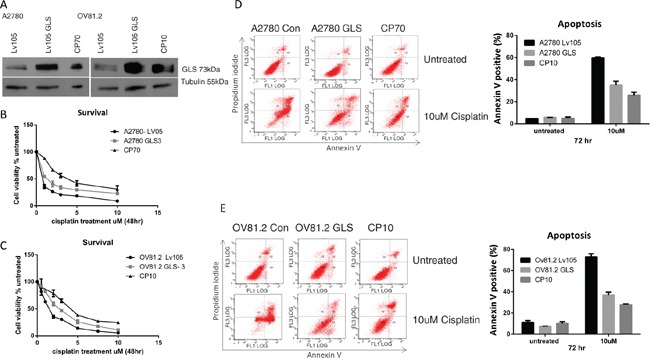
GLS overexpression confers cisplatin resistance **A.** Western blot confirms over expression of glutaminase (GLS) in A2780 and OV81.2 cells similar to the expression level found in the resistant cell lines. **B.** A2780 GLS over expressing cells and A2780 expressing control LV05 vector and **C.** OV81.2 GLS over expressing cell and OV81.2 expressing control LV05 vector were treated with increasing concentrations of cisplatin. After 48 hr treatment cell viability was assessed by Vi-CELL trypan blue counter. Experiments were performed in duplicate and repeated 3 times. Graphs present mean (dots) and SD (bars; n=6) of percentage viable cells relative to untreated. Apoptosis was measured using annexin V staining in **D.** A2780 expressing control LV05 vector, A2780 GLS over expressing cells and CP70 cells, and **E.** OV81.2 expressing control LV05 vector, OV81.2 GLS over expressing cells and CP10 cells. Cells were incubated with and without cisplatin (10uM) for 72 hr. Experiments were performed in duplicate and repeated 3 times. Graphs present mean (boxes) and SD (bars; n=6) of total apoptosis. Total apoptosis is the sum of the percentage of annexin V only and annexin V / propidium iodide stained cells.

To evaluate the effect of different levels of glutaminase (GLS) expression on cisplatin resistance we over-expressed GLS in the cisplatin sensitive A2780 and OV81.2 cell lines. Western blot analysis confirmed a significantly higher glutaminase expression in the GLS transfected cells compared to empty vector control (Figure 4A), and this over-expression is comparable to that found in the corresponding cisplatin resistant cell lines. Assessment of cell viability in the presence of increasing concentrations of cisplatin showed that a higher expression of glutaminase resulted in an increasing resistance to cisplatin compared to the control transfected cells (Figure 4B and 4C). This resulted in a significantly higher IC50 value for cisplatin in the glutaminase over-expressing cells compared to the transfected controls (A2780-control, 0.54uM+/−0.1; A2780-GLS, 1.18+/−0.04 (p<0.001) and OV81.2-control, 1.5+/−0.15; OV81.2-GLS 2.2+/−0.2 (p<0.001)). To determine if the increased resistance to cisplatin in the glutaminase over-expressing cell lines was mediated by reduced apoptosis we assessed the percentage of annexin V positive apoptotic cells. Glutaminase over-expressing cells had significantly reduced apoptosis in the presence of cisplatin treatment compared to the control cells (60% and 73% in A2780-control and OV81.2-control compared to 35% and 36% in the A2780-GLS and OV81.2-GLS, respectively) (Figure 4D and 4E). The reduced apoptosis upon cisplatin treatment in the glutaminase over-expressing cells was similar to the level of apoptosis seen in the corresponding resistant cell lines (Figure 4D and 4E).

**Figure 5:**
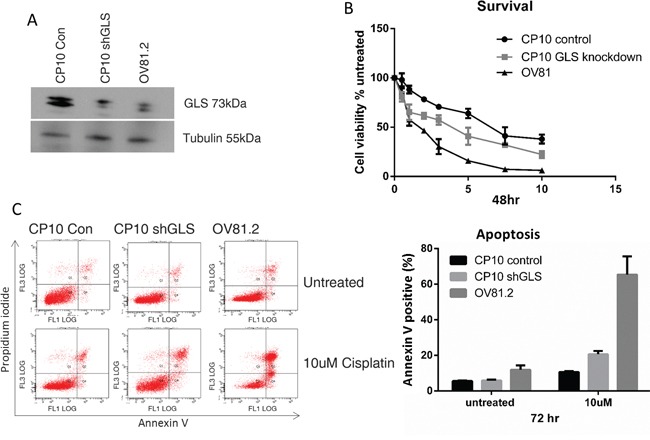
Loss of GLS expression re-sensitizes resistant cells to cisplatin **A.** Western blot confirms reduced expression of glutaminase (GLS) in CP10 shGLS similar to the expression level found in the sensitive cell lines. **B.** CP10 shGLS, CP10 shRNA control and OV81.2 were treated with increasing concentrations of cisplatin. After 48 hr treatment cell viability was assessed by Vi-CELL trypan blue counter. Experiments were performed in duplicate and repeated 3 times. Graphs present mean (dots) and SD (bars; n=6) of percentage viable cells relative to untreated. **C.** Apoptosis was measured using annexin V staining in CP10 shGLS, CP10 shRNA control and OV81.2. Cells were incubated with and without cisplatin (10uM) for 72 hr. Experiments were performed in duplicate and repeated 3 times. Graphs present mean (boxes) and SD (bars; n=6) of total apoptosis. Total apoptosis is the sum of the percentage of annexin V only and annexin V / propidium iodide stained cells.

The role of glutaminase in cisplatin resistance was further investigated by suppressing the expression of GLS using shRNA. CP10 cells were transduced with a lentivirus containing shRNA against GLS (shGLS) or a lentivirus containing a control shRNA. Immunoblot analysis confirmed that the GLS shRNA effectively reduced glutaminase protein expression in resistant cells, which was comparable to the level in the cisplatin sensitive cell lines (Figure 5A). This level of reduction lead to a statistically significant increase in cisplatin sensitivity compared to the control shRNA cells (p<0.001, Figure 5B). We further assessed if the increased sensitivity to cisplatin in the glutaminase knockdown cells was due to increased apoptosis. This was confirmed by the increased annexin V positive cells in the CP10 shGLS cells in the presence of cisplatin treatment compared to the control cells (20% in CP10 shGLS compared to 10% in the CP10 shRNA control (Figure 5C).

### Inhibition of glutaminase using BPTES synergizes with cisplatin treatment

**Figure 6:**
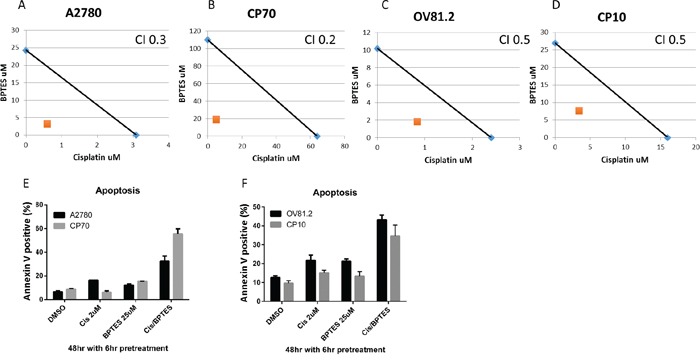
BPTES synergizes with cisplatin Isobologram analysis for **A.** A2780, **B.** CP70, **C.** OV81.2 and **D.** CP10 by combination of cisplatin and glutaminase inhibitor BPTES. The IC_50_ values of each drug are plotted on the x- and y-axis; the solid line represents the additive effect, whereas the point representing the concentrations of the combinations resulting in 50% cell viability is on the left indicating synergism. Combination index (CI) is less than 1 indicating synergism. Apoptotic induction in **E.** A2780 and CP70, **F.** OV81.2 and CP10 treated with DMSO, cisplatin, BPTES and combination. Experiments were performed in duplicate and repeated 3 times. Total apoptosis is the sum of the percentage of annexin V only and annexin V / propidium iodide stained cells.

The increased glutamine dependency of platinum resistant cells correlates with the upregulated glutamine utilization compared to cisplatin sensitive cell lines. Coupled to the observation that cisplatin treatment also induces glutamine uptake in sensitive cell lines, we therefore assessed if inhibiting glutaminase (GLS) with BPTES improves the therapeutic effectiveness of cisplatin treatment and is re-sensitizing drug resistant cells to the toxic effects of cisplatin. BPTES is a potent and selective allosteric inhibitor against both splice variants of the GLS gene, GAG and KGA, but not the hepatic form of glutaminase encoded by the GLS2 gene [23, 27]. In our paired cisplatin sensitive and resistant cell lines, isobologram and combination index (CI) analysis of BPTES and cisplatin combination revealed that these drugs work in a synergistic manner in all cell lines tested (CI range from 0.2-0.5, p<0.01 vs. either alone, Figure 6A-6D). This synergistic interaction resulted in increased apoptosis in the BPTES and cisplatin combination compared to single treatments (p<0.01, Figure 6E and 6F). These results support the approach of targeting the glutamine metabolism in addition to cisplatin treatment to improve therapeutic effectiveness in ovarian cancer.

### Higher glutaminase gene expression correlates with reduced survival of ovarian cancer patients

**Figure 7:**
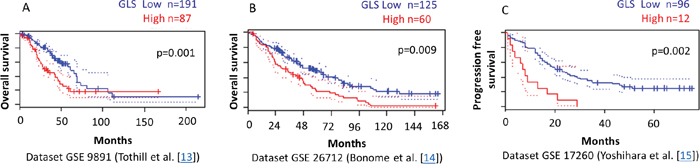
Higher GLS gene expression correlates with reduced survival of ovarian cancer patients PrognoScan database-based Kaplan-Meier analysis of three independent cohorts including 287, 185, and 108 ovarian cancer patients, respectively. Higher levels of GLS (red) are significantly correlated with reduced overall survival **A, B.** and reduced progression free survival **C.** compared to low GLS levels (blue) (p<0.01). This analysis was based on the PrognoScan database (http://www.prognoscan.org/) using the publicly available Gene Expression Omnibus (http://www.ncbi.nlm.nih.gov/geo) with the accession numbers GSE 9891, GSE 26712, and GSE 17260 [13–15, 28].

To validate the clinical relevance of our findings, we analyzed the relationship between glutaminase and glutamine transporter ASCT2 expression levels and outcome of ovarian cancer patients using the PrognoScan database [28]. In three independent publicly available microarray datasets of ovarian cancer patients [13–15], we found a significant correlation between higher levels of glutaminase gene expression and reduced progression free and overall survival (Figure 7). No significant correlation between ASCT2 and patient outcome was observed.

## DISCUSSION

Current platinum-based chemotherapy in ovarian cancer falls short due to the common development of drug resistance. This is often related to reduced platinum accumulation in ovarian cancer cells, linked to increased expression of multidrug resistance associated transporters [29]. In ovarian cancer, platinum resistance has also been associated with an increase in stem-like properties mediated by upregulated Wnt signaling [24] and differential expression of microRNAs such as miR-181a [30]. More recently several studies have highlighted the importance of altered glucose or fatty acid metabolism in mediating resistance to chemotherapy or targeted therapies [31–38]. Despite increased knowledge about the biology of platinum resistance, improved therapy in ovarian cancer has been lacking due to the complexity of the mechanisms involved and cross-talk and redundancy of governing signaling pathways [39, 40].

Within cell signaling pathways, the c-Myc transcription factor is a main regulator of energy metabolism and is often amplified or overexpressed in many types of cancers including ovarian cancer [8, 41]. Studies have demonstrated that c-Myc triggers cellular dependency on glutamine to feed the TCA cycle and increases anabolic pathways [8, 17, 25]. c-Myc expression has also been associated with platinum resistance in various cancer types [42–45] for example via repression of c-MYC inhibitor bridging integrator 1 [44] or increased platinum accumulation in cells [43]. Reyes-Gonzalez et al. recently reported shorter disease-free and overall survival in ovarian cancer patients with higher levels of c-Myc gene expression [45]. The group demonstrated that therapeutic siRNA-mediated silencing of c-Myc resulted in a significant reduction in tumor growth and induction of cell cycle arrest and apoptosis in ovarian cancer cell lines and murine xenograft models [45]. Our results suggest a potential additional mechanism of c-Myc induced platinum resistance in ovarian cancer through increased cellular utilization of glutamine. We have shown that the c-Myc expression is upregulated in platinum resistant compared to sensitive ovarian cancer cell lines. In addition, we found an upregulation of c-Myc in platinum sensitive cell lines during platinum treatment. c-Myc has been shown to regulate glutaminolysis by directly activating the expression of genes involved in glutamine metabolism such as the amino-acid transporter ASCT-2 and glutaminase (GLS) [7, 8] as well as regulating GLS through post-transcriptional mechanisms by repressing the transcription of miR-23a/b [26]. Consistent with this, we demonstrated that the effective inhibition of c-Myc with 10058-F4 in human ovarian cancer cell lines correlated with reduced glutaminase and ASCT-2 protein levels and subsequently reduced radiolabeled glutamine uptake.

A key finding of our study is the glutamine dependency of platinum resistant ovarian cancer cell lines. Platinum resistant cells were approximately 4-times more sensitive to glutamine deprivation compared to sensitive cells. In addition, platinum resistant cells were characterized by upregulated expression of important proteins in the glutamine metabolic pathway including glutamine transporter ASCT2 and glutaminase (GLS) (Figure 1C). We also found increased glutamine uptake and glutamine dependent TCA oxidative phosphorylation in platinum resistant compared to sensitive ovarian cancer cells. Underscoring the clinical significance of these findings, high glutaminase gene expression correlated with poor patient outcome in three independent publicly available microarray datasets of ovarian patients which we analyzed through the PrognoScan database (Figure 7) [13–15, 28]. Furthermore, both GLS and ASCT2 expression were increased in platinum sensitive cell lines treated with platinum, showing upregulated glutamine metabolism as an early event in response to platinum treatment.

To further evaluate the effect of different levels of glutaminase (GLS) expression on resistance to platinum we suppressed the expression of GLS using shRNA in platinum resistant cells and over-expressed GLS in platinum sensitive cell lines. Targeted inhibition of GLS resulted in a significant increase in platinum sensitivity compared to the control shRNA cells. Conversely, enhanced expression of GLS resulted in increased platinum resistance compared to the control transfected cells and significantly reduced apoptosis in the presence of platinum. Intriguingly, an increased glutamine dependency was recently linked to enhanced *in vitro* migratory and invasive capabilities in a panel of eight ovarian cancer cell lines [46]. Using transwell invasion and wound-healing assays, the authors showed that glutamine independent cell lines OVCAR3, IGROV1 and OVCA429 had low invasive capacities, whereas highly glutamine dependent SKOV3, SKOV3ip and Hey8 ovarian cancer cell lines had highest invasive capacities. In line with their levels of glutamine dependence, SKOV3 had a 4-fold higher expression of glutaminase (GLS1) compared to OVCAR3. Using *in-vivo* xenograft tumor models, GLS1 gene silencing by siRNA significantly reduced both tumor weight and tumor volume in Gln-dependent SKOV3ip1 tumor-bearing animals, whereas it did not affect the volume of Gln-independent IGROV1 tumors. In addition, when analyzing tissues of 139 ovarian cancer patients by immunohistochemistry, the group found an association between higher protein expression of GLS1 and reduced overall survival [46].

In this present study we uncover that the therapeutic inhibition of glutamine metabolism using glutaminase (GLS) inhibitors reversed platinum resistance and demonstrated synergistic efficacy in combination with cisplatin chemotherapy. BPTES (bis-2-(5-phenylacetamido-1,2,4-thiadiazol-2-yl)ethyl sulfide) is a potent and selective allosteric inhibitor against both splice variants of the GLS gene, GAG and KGA, but not the hepatic form of glutaminase encoded by the GLS2 gene, thus avoiding hepatic toxicity [23, 27]. The combination of platinum and the GLS inhibitor BPTES was markedly more effective than either agent alone in cisplatin sensitive or resistant human ovarian cancer cell lines (Figure 6A-6D). Of note, for the synergistic interaction, BPTES was required to be added several hours prior to platinum treatment and the clinical implications of this observation are not yet known. The combination treatment had a profound effect on cell survival, inducing apoptotic death and overcoming platinum resistance. A similar glutaminase inhibitor, CB-839 (Calithera Biosciences, Inc., South San Francisco, CA) was recently shown to display significant antitumor activity in two xenograft models of triple negative and basal-like HER2-positive breast cancer as a single agent and in combination with paclitaxel chemotherapy [16]. Further studies are needed to determine whether the observed synergistic effect of glutaminase inhibition in ovarian cancer is specific to platinum chemotherapy. Our novel finding that targeting glutamine metabolism can reduce or reverse platinum resistance has a high potential for clinical translation, since oral glutaminase inhibitors such as CB-839 are currently being evaluated in Phase 1 clinical trials in patients with advanced solid tumors (NCT02071862; ClinicalTrails.gov) and hematological malignancies (NCT02071888; ClinicalTrails.gov).

An important strength of our work is the utilization of two complementary ovarian cancer cell lines. We used the A2780/CP70 paired platinum sensitive and resistant cell line and also assessed a human primary cell line isolated from a patient with newly diagnosed high grade serous ovarian cancer (OV81.2). From this cell line we generated *in vitro* a platinum resistant cell line (CP10). These cell lines combine the advantages of I) utilizing a more artificial but long established and well-characterized model system of platinum resistant ovarian cancer (A2780/CP70) with the advantages of II) using a novel primary patient-derived cell line. Furthermore, these two cell lines represent the two most common histologic subtypes of human ovarian cancer, high-grade serous (OV81.2) and endometrioid (A2780; [47, 48]) which together account for more than 90% of human ovarian cancer cases, thereby making our results highly clinically relevant.

In conclusion our work provides the first evidence for increased glutamine dependency of platinum resistant ovarian cancer cells and demonstrates that targeting glutamine metabolism together with platinum based treatment offers a potential therapeutic strategy particularly in platinum resistant ovarian cancer. Nevertheless, cancer cells can readily switch energy sources [17] and utilize compensatory pathways. Therefore, combinations of inhibitors that target different metabolic pathways together with platinum chemotherapy may provide a more effective cytotoxic combination while reducing the development of resistance. Changes in tumor metabolic activity can be non-invasively assessed via positron emission tomography (PET). PET biomarkers for both, glucose and glutamine metabolism are either in routine clinical use or currently being evaluated in clinical trials [49–51, 52–56]. In future clinical trials, non-invasive PET imaging of glucose and glutamine metabolic pathways could be used to visualize net changes in a tumor's energy consumption and detect a potential switch in tumor metabolic activity early during therapy. A molecular imaging guided approach to metabolic pathway inhibition could provide a rationale for individualized combinations of metabolic pathway inhibitors and chemotherapy and potentially improve the outcome of ovarian cancer patients.

Further *in vivo* studies of targeting glutamine metabolism in combination with platinum chemotherapy are warranted and may set the stage for subsequent clinical trials to exploit the glutamine dependency of platinum resistant ovarian cancer.

## MATERIALS AND METHODS

### Reagents

Cisplatin, glutaminase inhibitor BPTES, and c-Myc inhibitor 10058-F4 were purchased from Sigma-Aldrich (St. Louis, MO, USA).

### Cell lines

Human ovarian cancer paired platinum sensitive and resistant cell lines A2780 (cisplatin sensitive) and CP70 (isogenic cisplatin resistant to A2780), and OV81.2 (cisplatin sensitive) and CP10 (isogenic cisplatin resistant to OV81.2) were provided by Dr. A. DiFeo. The OV81.2 cell line was previously generated by Dr. DiFeo from the ascites of a patient diagnosed with high grade serous ovarian cancer who was platinum sensitive. The platinum resistant derivative of OV81.2, namely, CP10, was generated by propagating OV81.2 in the presence of 2.5μM cisplatin for 10 passages *in vitro* as previously described [24]. After selection for acquired platinum resistance CP10 cells are grown in the absence of cisplatin. Cell lines were maintained in high glucose Dulbecco's modified Eagles medium (DMEM) supplemented with 10% fetal bovine serum. Glucose and glutamine free medium supplemented with 2% dialyzed fetal bovine serum was used in the *in vitro* uptake assays.

### Lentiviral transduction

GLS over expression was achieved by stable transduction with Lentivirus, using the vector containing the sequence of GLS1 in the pEZ-Lv105 vector (EX-H0487-Lv105-10; GeneCopoeia, Rockville, MD, USA). Control plasmid consisted of pEX-NEG-Lv105. Gene knockdown was achieved by stable transduction with Lentivirus, using the vector psi-LVRH1GP containing a GLS specific short hairpin RNA or control sequence (HSH007722-LVRH1GP; GeneCopoeia, Rockville, MD, USA). The transduced cells were enriched by puromycin (3ug/ml) selection as well as GFP in the case of knockdown. Protein over expression or knockdown was confirmed by Western blot.

### Cell viability assay

The growth inhibitory effects of cisplatin, BPTES and 10058-F4 were measured using the Vi-CELL trypan blue exclusion counter (Beckman Coulter, Indianapolis, IN, USA). Cells were plated out in 6 well plates (Corning, NY, USA) at 1 × 10^5^ cells per well the night before drug treatment. Cells were then incubated with different drug concentrations or combinations as stated for 48 hr. Viable cell numbers from triplicate wells were determined using the Vi-CELL counter, and IC_50_ values were calculated using GraphPad Prism Software Version 5 (GraphPad Software Inc., La Jolla, CA, USA) and plotted in dose response curves.

### Annexin V staining

Cells (1 × 10^5^) were seeded in 6 well plates and allowed to adhere overnight before incubation in the presence or absence of cisplatin, BPTES or combinations for 48 or 72 hr. Cells were detached using versene, washed in PBS and resuspended in 1X annexin buffer. FITC annexin V and PI (BD Biosciences, San Jose, CA, USA) were added and incubated for 15 minutes in the dark. Annexin V Alexa Fluor 647 was used for GFP expressing cell lines. The cells were analyzed using the BD LSR II flow cytometer (BD Biosciences, San Jose, CA, USA).

### Protein expression

Protein was isolated from cells using lysis buffer (20 mM Tris pH 8, 200 mM NaCl, 1 μm EDTA pH 8 0.5% Np40 and 10% glycerol). Lysates were standardized for protein content and 20 μg of total protein was separated by a gradient 4-20% SDS-polyacrylamide gel (Mini-PROTEAN TGX, Bio-Rad, CA, USA) electrophoresis and transferred to PVDF membranes using the iBlot system (Invitrogen, ThermoFisher Scientific, Grand Island, NY, USA). The membranes were blocked and primary antibody was diluted 1:1000 in 3% BSA in TBS containing 0.01% Tween 20 (TBST) and incubated overnight at 4°C. The following day, blots were washed in TBST buffer and incubated with goat anti-rabbit horseradish peroxidase-conjugated secondary antibody (Dako, Carpinteria, CA, USA) for 1 hr at room temperature. After washing in TBST buffer (three times, 10 min per wash) the immunoreactive proteins were visualized using ECL detection reagent (Amersham, GE Healthcare Biosciences, Pittsburgh, PA, USA). The antibodies used were anti-ASCT2 (V501, Cell Signaling, Beverly, MA, USA) and anti-c-Myc (D84C12, Cell Signaling, Beverly, MA, USA), anti-glutaminase (Abcam, Cambridge, MA, USA) and anti-β-tubulin (Santa Cruz Biotechnology, Dallas, TX, USA).

### *In vitro* tracer uptake assays

Cells were plated at 1 × 10^5^ cells per well the evening before the experiment. The next day cells were incubated with drug for 24hr (c-Myc inhibition) or 48hr (cisplatin). On the day of the assay, the drug-containing media was removed and cells incubated in glucose/glutamine free media supplemented with 2% dialyzed fetal bovine serum containing [C-14]DG and [H-3]GLN at 1 μCi/ml to each well and incubated for 20 mins. Cells were then washed twice in ice cold PBS, harvested with trypsin, and viable cell numbers determined using the Vi-CELL counter. The cell lysates were collected and added to tubes containing 2 ml of scintillation fluid and the cell associated [C-14] and [H-3] radioactivity was measured using the β scintillation counter (LKB Instruments, Mount Waverly, Victoria, AU). Radioactivity uptake is expressed as nanomoles uptake of tracer per 10 000 live cells and expressed relative to untreated controls. All experiments were performed in triplicate and repeated 3 times.

### Oxygen consumption rate (OCR)

The rate of change of dissolved O_2_ (oxygen consumption rate, OCR) was determined using the XF24 Analyzer (Seahorse Bioscience, MA, USA). Drug-treated cells were harvested and 100,000 cells were plated out on the cellTak (BD Biosciences, San Jose, CA, USA) treated microplates. The rate of change of dissolved O_2_ in the medium immediately surrounding the cells was measured using the XF24 Analyzer. The contribution of glutamine in oxidative phosphorylation was assessed using specific inhibitors to glutamine metabolism or stable over expression or knockdown of GLS.

### PrognoScan analysis

Using the PrognoScan database (http://www.prognoscan.org/) and publicly available Gene Expression Omnibus (http://www.ncbi.nlm.nih.gov/geo) datasets with the accession numbers GSE 9891, GSE 26712, and GSE 17260, the relationship between GLS expression levels and overall or progression free survival rates was evaluated in ovarian cancer patients [13–15, 28]. Patients were stratified into two groups according to their intratumoral GLS expression at various cutoffs, and the cutoff point yielding the most significant survival difference by log-rank test is presented in the Kaplan Meier analysis.

### Statistical analysis

Quantitative parameters are reported as mean values and standard deviation (SD). Differences between treatment groups or experimental factors were assessed by analysis of variance (ANOVA) using GraphPad Prism Software Version 5 (GraphPad Software Inc., La Jolla, CA, USA) at a two-sided 5% significance level.
